# *Clogmia albipunctata* fails to induce true intestinal myiasis: Evidence from *in vitro* and *in vivo* digestive models

**DOI:** 10.1371/journal.pone.0356742

**Published:** 2026-08-21

**Authors:** Huahan Zhan, Linghong Zhang, Lu Ge, Yanli Chen, Peiyi Zhang, Yumeng Jiao, Xiaocheng Luo, Hui Xia, Qiang Fang, Zhiyong Tao

**Affiliations:** 1 Anhui Key Laboratory of Infection and Immunity, Bengbu Medical University, Bengbu, Anhui, China; 2 First School of Clinical Medicine, Bengbu Medical University, Bengbu, Anhui, China; 3 Department of Parasitology, School of Basic Medical Sciences, Bengbu Medical University, Bengbu, Anhui, China; 4 Department of Clinical Laboratory, The Fourth People’s Hospital of Nanning, Nanning, Guangxi, China; 5 School of Laboratory Medicine, Bengbu Medical University, Bengbu, Anhui, China; World Health Organization, Regional Office for South-East Asia, INDIA

## Abstract

**Objective:**

*Clogmia albipunctata* (*C. albipunctata*) is a synanthropic moth fly frequently found in humid human environments. Larvae of this species have been reported in suspected cases of intestinal myiasis, but whether they can establish true gastrointestinal infestation remains uncertain. This study evaluated the survival, development, and colonization potential of *C. albipunctata* using *in vitro* digestive models and murine oral gavage models.

**Methods:**

Wild-caught moth flies were reared in artificial climate chambers to establish laboratory colonies. Species identification was subsequently performed through morphological characterization and molecular analysis. The *in vitro* digestion model exposed *C. albipunctata* eggs and larvae to simulated gastrointestinal fluids, and their survival was monitored. For *in vivo* assessment, BALB/c mice were orally gavaged with viable eggs. Feces were examined for seven consecutive days, and gastrointestinal tissues were evaluated by time-point necropsy and histological examination.

**Results:**

Laboratory colonization of *C. albipunctata* was successfully achieved, and the species identity was confirmed. The *in vitro* digestion model demonstrated that standard simulated gastric fluid acts as a lethal barrier. Specifically, egg hatchability decreased from 76.80% ± 3.57% in controls to 0% after digestion, and larval mortality increased from 19.17% ± 3.01% to 99.17% ± 0.83% after simulated gastric exposure. While simulated postprandial weakly acidic conditions (pH 2.0–5.0) partially attenuated this lethality, sequential intestinal fluid exposure induced further mortality. *In vivo*, the oral gavage of viable eggs and live first-instar larvae in mice resulted in no fecal shedding, tissue injury, or pathological colonization in the gastrointestinal tract.

**Conclusions:**

These findings indicate that healthy murine gastrointestinal conditions and simulated human digestive fluids are highly unfavorable for the survival and colonization of *C. albipunctata* eggs and larvae. The results support the interpretation that many suspected clinical cases may represent sample contamination or pseudomyiasis rather than true intestinal myiasis.

## Introduction

Moth flies are dipteran insects that undergo complete metamorphosis, including egg, larval, pupal, and adult stages [1]. *Clogmia albipunctata* (*C. albipunctata*) is a common synanthropic moth fly with a broad distribution, particularly in tropical and subtropical regions [2,3]. Its larvae feed on decaying organic matter and frequently develop in bathrooms, kitchens, drains, garbage sites, and sewage systems [2–5]. Adult moth flies do not bite or suck blood, but their frequent contact with human environments allows them to act as potential mechanical carriers of microorganisms [6–9].

In recent years, *C. albipunctata* has been increasingly reported in suspected cases of human myiasis involving the gastrointestinal, urinary, genital, nasal, or other systems [10–24]. For suspected intestinal myiasis, larvae are usually detected in fecal samples, whereas direct evidence of larval invasion, development, or colonization within the intestine is rarely available. Therefore, the clinical interpretation of these findings remains controversial. Larvae found in feces may represent true intestinal myiasis, but they may also result from pseudomyiasis, accidental passage through the digestive tract, or contamination of fecal samples after defecation [2,25]. This distinction is clinically important because overdiagnosis may lead to unnecessary antiparasitic treatment and patient anxiety.

The digestive tract is an open system that can be exposed to environmental eggs or larvae through contaminated food or water. However, ingested arthropod stages must survive gastric acidity, digestive enzymes, bile salts, intestinal transit, and host immune defenses before they can establish true intestinal colonization. Whether *C. albipunctata* eggs or larvae can overcome these barriers has not been experimentally demonstrated.

*In vitro* digestion models based on standardized INFOGEST protocols are widely used to simulate human gastrointestinal conditions in food and pharmaceutical research [26–28]. To our knowledge, these models have not previously been applied to evaluate the intestinal pathogenic potential of moth flies. In the present study, we established a stable laboratory colony of *C. albipunctata*, confirmed species identity by morphology and COI barcoding, and assessed the survival of eggs and larvae under simulated gastrointestinal conditions. We further tested whether orally administered eggs or first-instar larvae could survive, be excreted, or colonize the digestive tract of BALB/c mice. This combined *in vitro* and *in vivo* approach was designed to clarify whether *C. albipunctata* can establish true intestinal myiasis under experimental conditions.

## Materials and methods

### Ethics statement and animals

This study was conducted in accordance with the National Guidelines for Experimental Animal Welfare of China and was approved by the Experimental Animal Management and Ethics Committee of Bengbu Medical University, China, under approval number 2023−216. All efforts were made to minimize animal suffering and to reduce the number of animals used.

Female BALB/c mice, 7 weeks old and weighing 18–22 g, were purchased from Changzhou Cavins Laboratory Animal Co., Ltd. (license no. SCXK Su 2021-0013). Mice were housed under specific-pathogen-free conditions at 22 ± 2°C, 50–60% relative humidity, with a 12 h light/12 h dark cycle. Standard laboratory chow and sterile water were provided ad libitum. Mice were acclimatized for 7 days before experiments.

Mice were monitored daily for general appearance, body weight, activity, food and water intake, diarrhea, abdominal distension, and signs of distress. Humane endpoints included weight loss exceeding 20%, severe lethargy, persistent diarrhea, inability to access food or water, or any signs of severe pain or distress. No animals reached humane endpoints before the scheduled sampling time points. At designated time points, mice were anesthetized with isoflurane using 3–5% for induction and 1–2% for maintenance. Euthanasia was performed by cervical dislocation under deep anesthesia, and death was confirmed by cessation of breathing and heartbeat before tissue collection.

### Laboratory colonization of *C. albipunctata*

**Fig 1:**
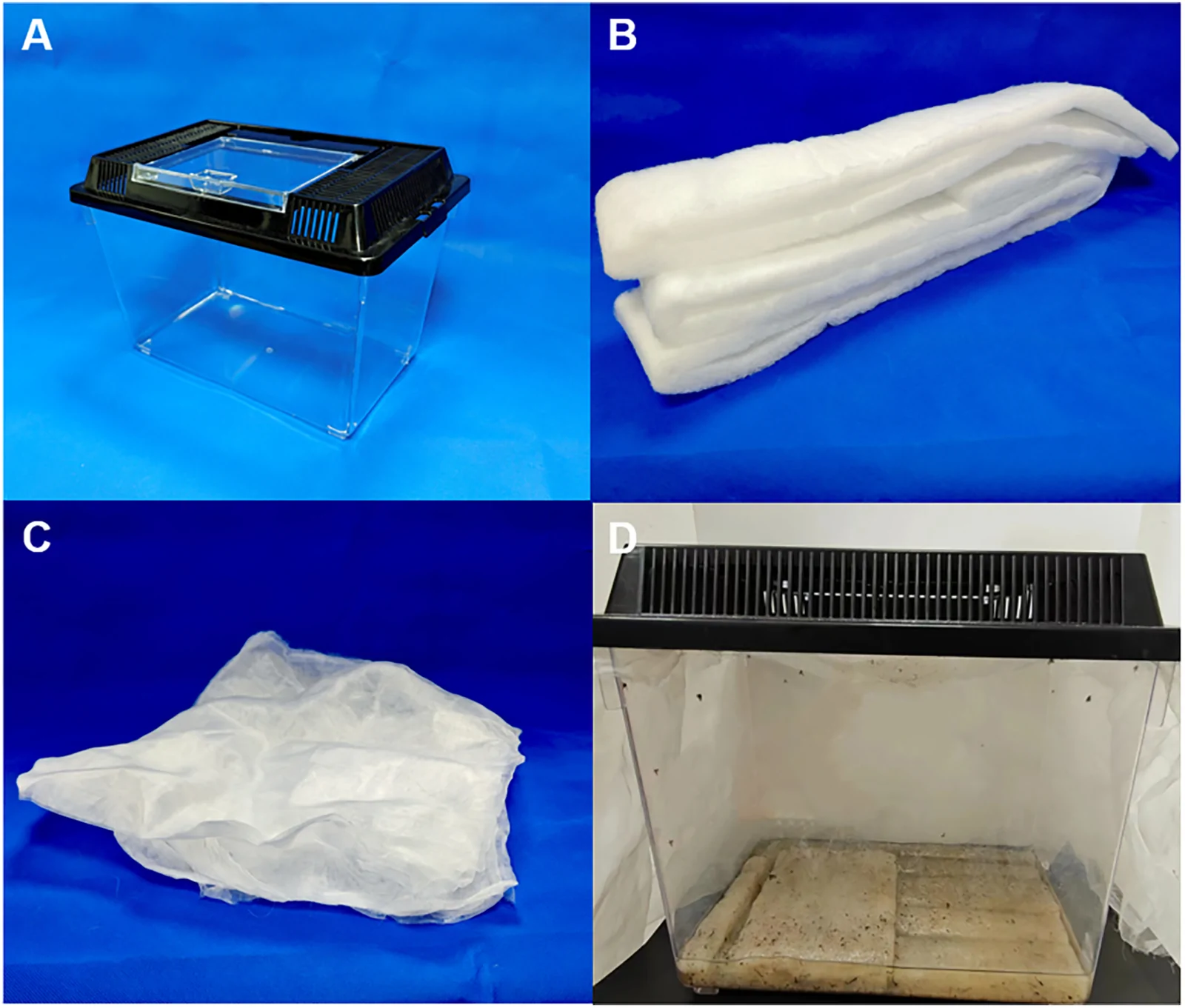
Containers and materials used for domestication. (A) Commercially available fish tank. (B) Filter pad. (C) Insect screen. (D) Mounted feeding device.

In accordance with the habits of *C. albipunctata* [1,4,29,30], field-caught *C. albipunctata* were collected from humid environments near Bengbu Medical University and used to establish a laboratory colony. A semi-natural breeding system was constructed in plastic breeding tanks containing aquarium filter pads as oviposition and pupation substrates (Fig 1). The tanks were covered with 100-mesh insect screen to prevent escape while allowing ventilation. A diet of ground insect carcasses was provided as the larval food source and was stored at 4°C before use.

Colonies were maintained in an artificial climate chamber at 28°C, 75% relative humidity, and a 16 h light/8 h dark photoperiod.

### Morphological observations of *C. albipunctata*

Eggs, larvae, pupae, and adults were collected from the laboratory colony for morphological observation, photography, and comparison with published descriptions of *C. albipunctata* [1,17,29]. For egg observation, 10 mL of culture fluid was aspirated from the breeding tank and centrifuged at 100 × g for 2 min. The supernatant was discarded, and the pellet was resuspended in sterile water or saline. Samples were transferred to glass slides, covered with coverslips, and examined under a light microscope.

Larvae and pupae with intact morphology were selected using fine forceps and observed under a stereomicroscope. Adult *C. albipunctata* were collected using an aspirator and briefly anesthetized with isoflurane vapor in a closed container for 2–3 s or until immobilized. Immobilized adults were placed on slides and examined under a stereomicroscope.

### Molecular characterization of *C. albipunctata*

Genomic DNA was extracted from laboratory-reared larvae using a blood/tissue/cell genomic DNA extraction kit (Tiangen, Beijing, China) according to the manufacturer’s instructions. Larvae were homogenized, filtered through a 70 μm cell strainer (Labgic, Beijing, China), and washed with PBS before DNA extraction. DNA was stored at −20°C until use.

The mitochondrial cytochrome c oxidase subunit I gene was amplified using the universal COI primers LCO1490 and HCO2198 (S1 Table). PCR conditions were adapted from established protocols and provided in S2 Table [7,13,31–33]. PCR products were examined by 2% agarose gel electrophoresis and purified for Sanger sequencing. Valid sequences were analyzed using BLASTn against the NCBI GenBank nucleotide database. Species identification was considered reliable when the sequence identity was greater than 97% with reference *C. albipunctata* sequences and query coverage was sufficient for barcode-level identification.

### Effects of changes in ambient temperature on the development of *C. albipunctata*

To evaluate the effect of mammalian physiological temperature on *C. albipunctata* development, eggs were incubated at 37°C and compared with eggs maintained under the routine insect-rearing temperature of 28°C. Adult *C. albipunctata* were transferred to oviposition chambers, and egg-containing suspensions were collected from the culture system. Samples were centrifuged at 100 × g for 5 min, and the pellet was resuspended in 1 mL ultrapure water. Egg numbers were counted microscopically at 100 × magnification.

For each temperature condition, six independent biological replicates were performed, with 200 eggs per replicate. Eggs were placed in filter paper-lined Petri dishes and incubated at either 28°C or 37°C under the same humidity and photoperiod conditions. Egg hatchability was calculated as the number of eggs that hatched into larvae divided by the total number of eggs × 100%. Larval survival was calculated as the number of first-instar larvae that survived and successfully pupated divided by the total number of larvae × 100%. Adult emergence was calculated as the number of pupae that successfully emerged as adults divided by the total number of pupae × 100%.

### Construction of an *in vitro* digestion model

A modified INFOGEST-based *in vitro* digestion model was used to assess the effects of simulated gastrointestinal fluids on *C. albipunctata* eggs and larvae [28,34–36]. The gastric phase was performed in simulated gastric fluid (SGF) at 37°C for 2 h, and the intestinal phase was performed in simulated intestinal fluid (SIF) at 37°C for 2.5 h using a temperature-controlled orbital shaker at 250 rpm. The final digestion system contained pepsin at 2000 U/mL during the gastric phase and pancreatin trypsin activity at 100 U/mL plus bile salts at 15 mg/mL during the intestinal phase. Detailed reagent compositions are provided in Table 1.

**Table 1:** Composition of the simulated digestion system for *in vitro* digestion experiments.

Digestion phase	Gastric	Intestinal	
Sample solution	15 mL suspension containing larvae/eggs^a^	15 mL suspension containing larvae/15 mL from gastric phase	
CaCl_2_ concentration (mM) in total digesta (final volume in milliliters at each digestion phase)	0.45	0.525	
CaCl_2_ (0.1 M) (mL)	0.135	0.0225^b^	
Enzymes	Pepsin	Trypsin in pancreatin	Bile salts
Enzyme activity (U/mL) or bile concentration (mg/mL) in total digesta	2,000 U/mL	100 U/mL	15 mg/mL
Activity of the enzyme used (U/mg), concentration (bile salt)	800−2,500 U/mg	250 U/mg	≥98%
Concentration of enzyme/bile solution (mg/mL)	20	20	200
Volume of enzyme/bile to be added (mL)	3.75	0.6	2.25
HCl (1 M) for pH adj.	Adjust to pH 1.5–2.0	–	
NaOH (1 M) for pH adj.	–	Adjust to pH 7.0	
HCl/H_2_O	Make up to final volume with reagent of equal pH		
Final volume (mL)	30	30	

The following reagents were used: pepsin (Labgic, Beijing, China), trypsin (Phygene, Fuzhou, China), bovine bile salts (Macklin, Shanghai, China). ^a^Suspensions were made by mixing eggs or larvae extracted from culture systems with ultrapure water (see the section “Effects of changes in ambient temperature on the development of *C. albipunctata*” for more information). ^b^This addition includes the amount added during gastric digestion, whereas the separate intestinal digestion model mentioned in the experimental design required additional CaCl_2_ solution to reach the concentration specified in this table.

Eggs and larvae were tested in parallel. For egg assays, suspensions containing 200 eggs/mL were used. For larval assays, each replicate contained 20 viable larvae. Control groups were treated with ultrapure water for the corresponding duration under identical temperature and shaking conditions. Unless otherwise stated, each *in vitro* experiment was performed with six independent biological replicates.

Egg hatchability was defined as the proportion of eggs from which larvae completely emerged from the eggshell. Larval survival was defined by intact morphology, spontaneous movement or movement after gentle tactile stimulation, and the ability to continue development. Larvae lacking movement after stimulation and showing disrupted morphology, rigidity, or collapse were recorded as dead.

### *In vitro* digestion of *C. albipunctata* larvae and eggs using SGF and SIF

To assess egg viability under simulated human gastrointestinal conditions, a sequential digestion protocol was employed. The gastric phase was simulated in a 30-mL digestion solution, continuously agitated in a temperature-controlled orbital shaker (37°C, 250 rpm). Digested suspensions were centrifuged (100 × g, 2 min, 25°C) to recover the eggs. To arrest residual enzymatic activity, the resulting pellet was washed twice with 30 mL of ultrapure water via successive centrifugation cycles. The final pellet was then gently resuspended in 30 mL of ultrapure water. For immediate viability assessment, a 15-mL aliquot of this suspension was seeded into Petri dishes containing a filter pad and maintained under routine incubation conditions (28°C). For the subsequent intestinal phase digestion, the remaining 15-mL fraction was centrifuged and reconstituted to a 30-mL volume using SIF diluted 1:1 (v/v) with ultrapure water. This gastrointestinal sequential digestion model allowed for the precise evaluation of post-gastric developmental competence.

Egg developmental outcomes were evaluated via hatching assays, with equal volumes of ultrapure water serving as the control.

Larval survival assays were conducted following digestion conditions identical to those used for eggs. Live larvae were randomly allocated into four experimental groups: (1) Untreated control group, (2) SGF treatment group, (3) SIF treatment group, and (4) SGF+SIF treatment group. The digestion protocols were performed exactly as described above, substituting the egg suspensions with live larval specimens when required.

To evaluate whether weakly acidic postprandial gastric conditions altered the survival of *C. albipunctata* eggs or larvae, SGF assays were conducted at pH 2.0, 2.5, 3.0, 3.5, 4.0, 4.5, and 5.0. Eggs and larvae were exposed to SGF at each pH value for 2 h at 37°C. After SGF treatment, samples were either assessed directly or transferred to SIF for an additional 2.5 h to simulate sequential gastric-intestinal exposure. Egg hatchability and larval mortality were calculated as described above. Each pH condition was tested in six independent biological replicates.

### Development of *C. albipunctata* in the digestive tract of mice

To evaluate the gastrointestinal colonization potential of *C. albipunctata*, BALB/c mice were orally gavaged with either viable eggs or viable first-instar larvae. For the egg-gavage group, each mouse received 0.5 mL of egg suspension containing 400 eggs/mL, corresponding to 200 eggs per mouse. For the larval-gavage group, each mouse received 200 viable first-instar larvae suspended in 0.5 mL sterile water. Control mice received 0.5 mL sterile water.

After gavage, feces were collected daily for seven consecutive days. Fecal samples were diluted with sterile saline and examined under a stereomicroscope or light microscope to detect eggs, larvae, pupae, or other developmental stages.

To avoid missing early degradation or excretion events, additional mice were euthanized at 6 h, 24 h, 48 h, 4 d, and 7 d after gavage. At each time point, 6 mice from the egg-gavage, larval-gavage, and control groups were examined. The esophagus, stomach, small intestine, and large intestine were dissected and inspected for obstruction, perforation, mucosal lesions, and visible insect developmental stages.

On day 7 after gavage, the esophagus, stomach, small intestine, and large intestine were collected from mice in the egg-gavage, larval-gavage, and control groups. Tissues were fixed in 4% paraformaldehyde, embedded in paraffin, and stained with hematoxylin and eosin. Sections were examined under a light microscope for epithelial disruption, mucosal ulceration, edema, hemorrhage, inflammatory cell infiltration, and the presence of insect structures.

### Statistical analysis

Statistical analyses were performed using GraphPad Prism 8.0. *In vitro* experiments were performed with six independent biological replicates unless otherwise stated. Proportional data, including egg hatchability, larval survival, larval mortality, and adult emergence rate, were calculated for each replicate before statistical analysis. All quantitative data are presented as the mean ± standard error of the mean (SEM). Comparisons between two groups were performed using an unpaired two-tailed Student’s t-test. Comparisons among multiple groups were performed using one-way ANOVA followed by Tukey’s multiple comparisons test. Categorical detection results in mouse feces or tissues were summarized descriptively because no developmental stages were detected. Differences were considered statistically significant at *p* < 0.05. In figures, * indicates *p* < 0.05, ** indicates *p* < 0.01, and *** indicates *p* < 0.001; different lowercase letters indicate statistically significant differences among groups at *p* < 0.05; different uppercase letters indicate statistically significant differences among groups at *p* < 0.01. All raw experimental data supporting the findings of this study are available in S2 File.

## Results

### Laboratory colonization of *C. albipunctata*

A stable laboratory colony of *C. albipunctata* was successfully established under semi-naturalized conditions. Adults mated and laid eggs on the sidewalls of breeding tanks and within the pores of aquarium filter pads. Eggs developed into larvae in the organic liquid substrate, larvae pupated in the filter pads or substrate, and adults subsequently emerged, completing the life cycle within approximately 3–5 weeks. The colony was maintained for more than 45 generations. Eggs and larvae used for the pathogenicity experiments were obtained from generations F23–F45.

### Morphological observations of *C. albipunctata*

**Fig 2:**
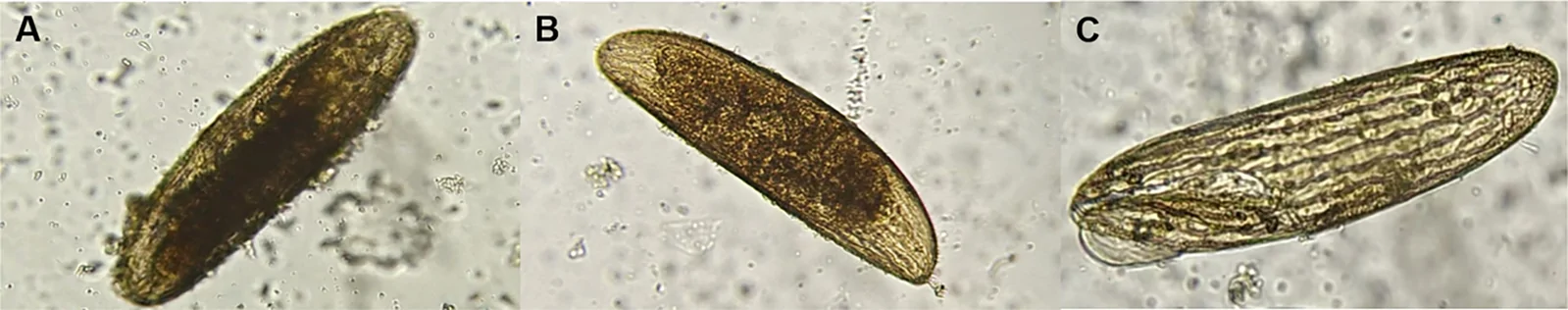
Morphology of *C. albipunctata* eggs at various time points. (A), (B), and (C) show three forms of *C. albipunctata* egg, where (C) shows an empty shell after larval hatching.

Samples from a *C. albipunctata* rearing chamber were collected, concentrated via centrifugation, and resuspended in saline for dilution. Fig 2 shows the fly eggs under a light microscope.

**Fig 3:**
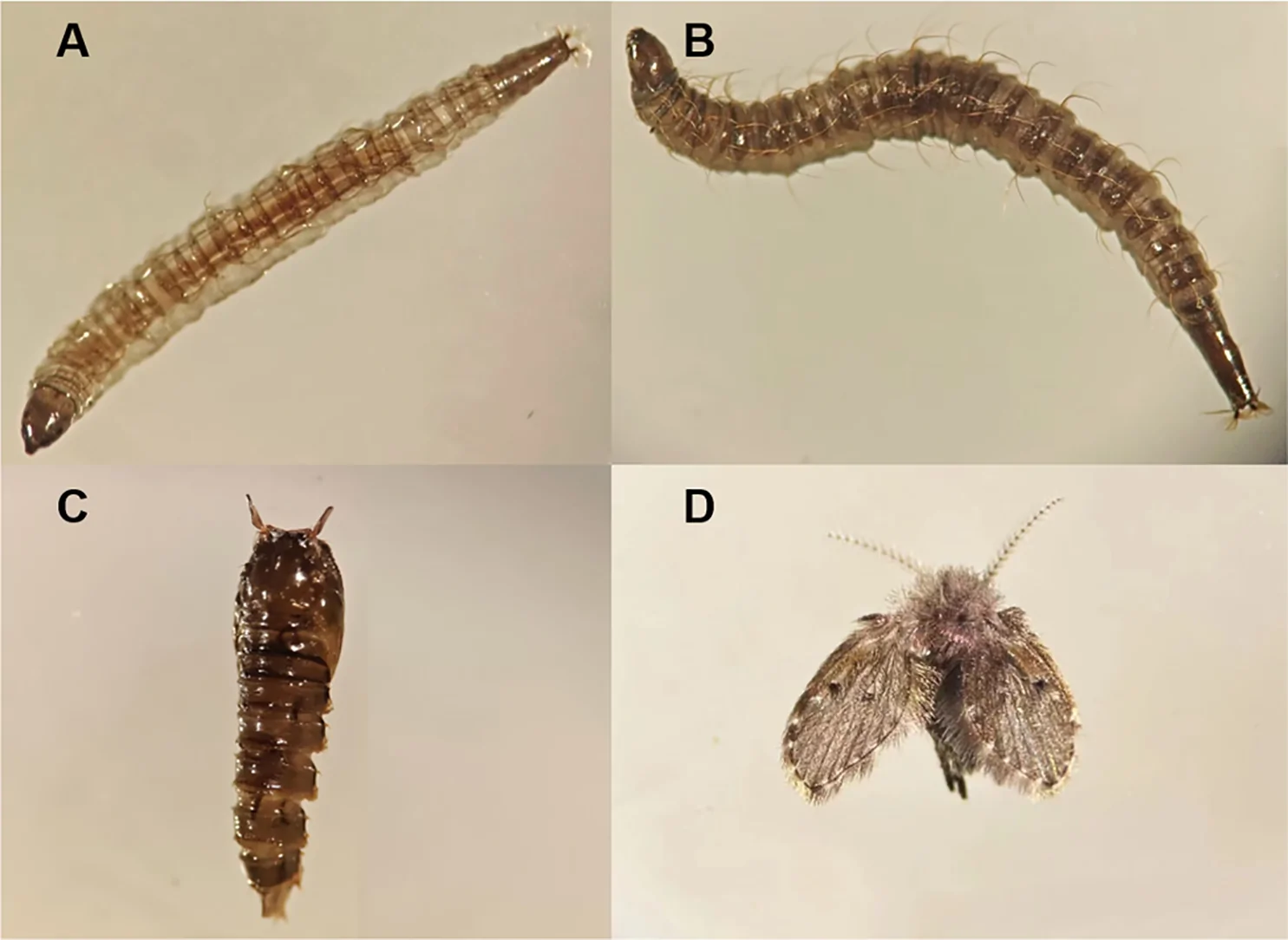
Morphology of larvae, pupae, and adults of *C. albipunctata.* (A) A low-instar larva. (B) A large instar larva. (C) A pupa. (D) An adult *C. albipunctata*.

Larvae, pupae, and adults in active condition and with complete morphology were selected from the rearing unit and observed under a stereomicroscope as live larvae and pupae and as adults anesthetized with isoflurane. Living larvae of *C. albipunctata* are shown in Fig 3A and B, the pupae are shown in Fig 3C, and the adults are shown in Fig 3D. Morphological observations revealed that the samples were in accordance with *C. albipunctata*.

### Molecular characterization of moth flies

**Fig 4:**
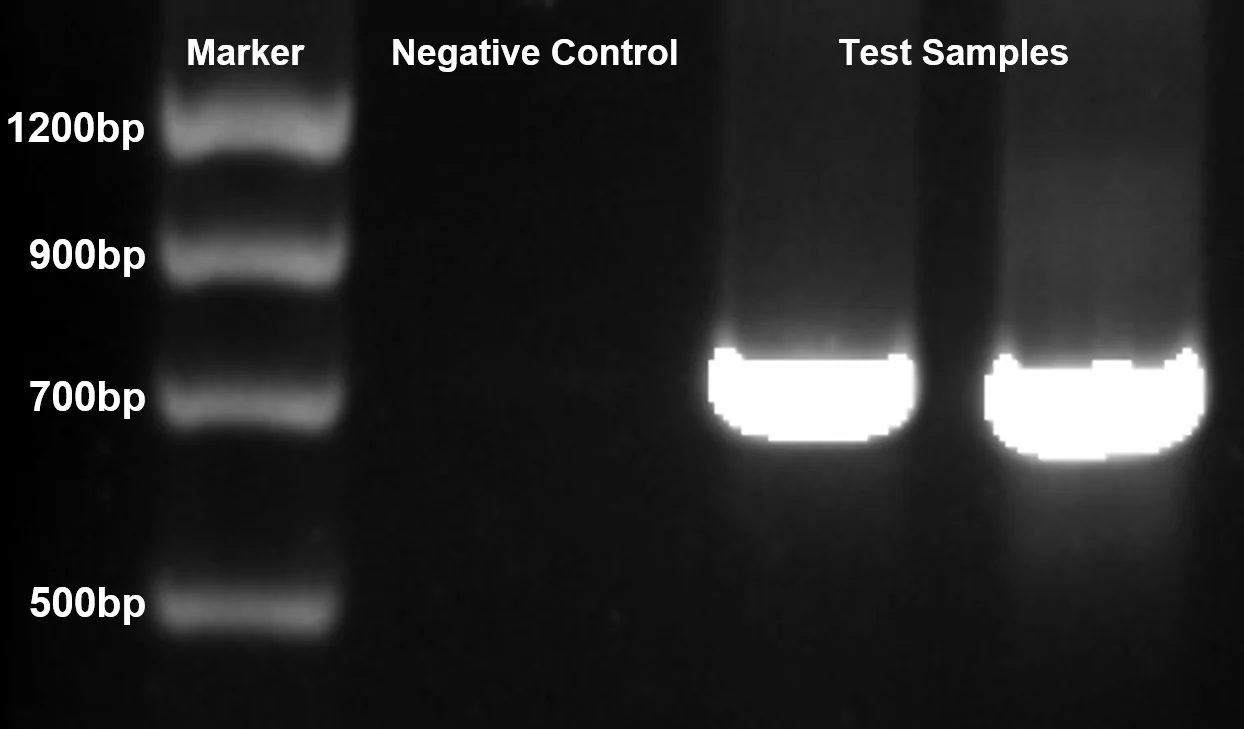
Agarose gel electrophoresis of COI gene PCR products.

COI fragments were successfully amplified from laboratory-reared larvae using universal barcoding primers. Agarose gel electrophoresis showed a single band of approximately 700 bp (Fig 4, the full-length gel is presented in S1 File). BLASTn analysis against the NCBI GenBank nucleotide database showed 100% identity and 98.25% query coverage with reference sequences of *C. albipunctata*. Together with morphological characteristics, these results confirmed that the laboratory colony was *C. albipunctata*.

### Effects of temperature on the development of *C. albipunctata*

**Fig 5:**
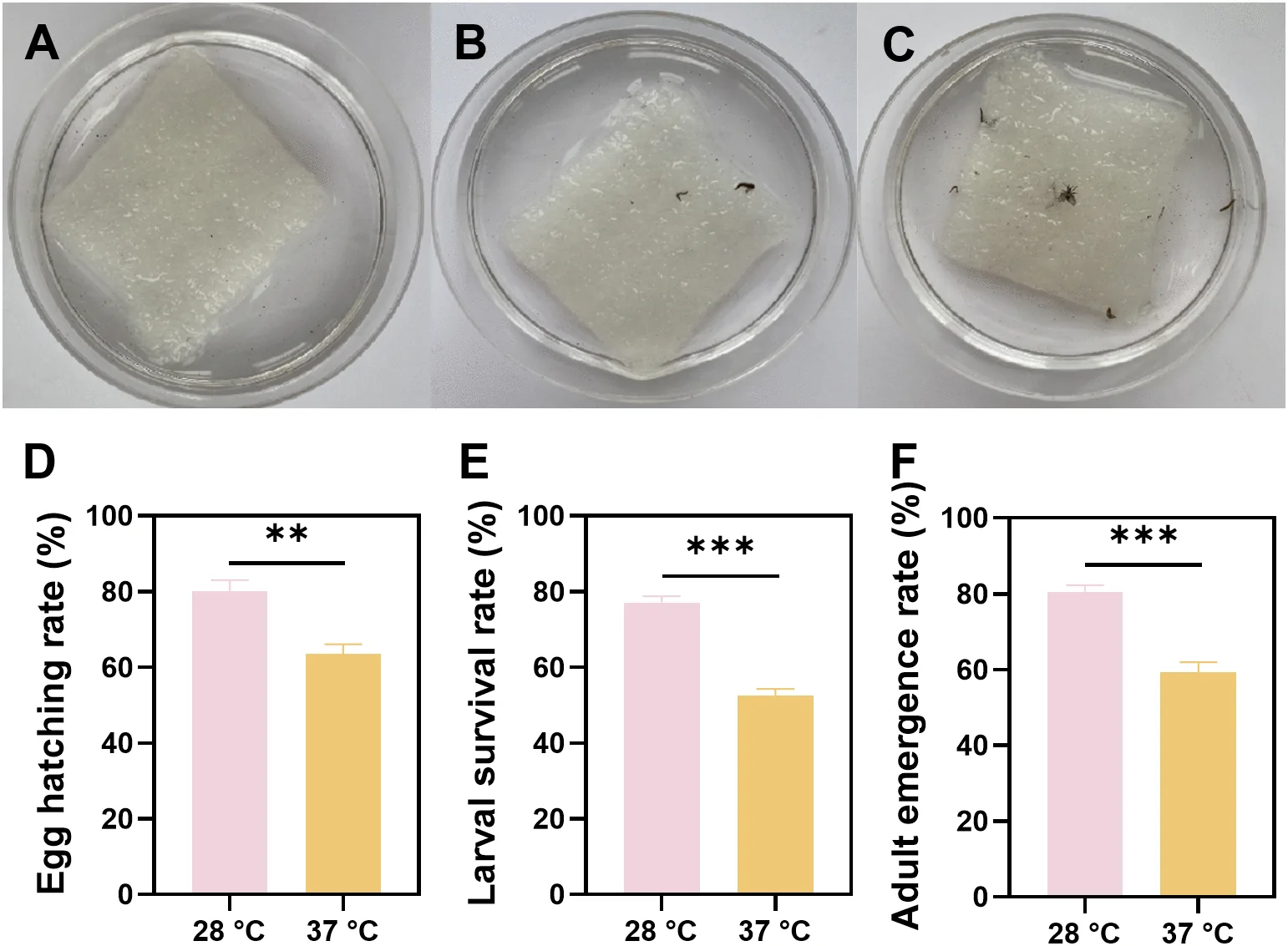
Development of *C. albipunctata* eggs at mammalian physiological temperature (37°C) compared with standard rearing temperature (28°C). (A) Unhatched *C. albipunctata* eggs at 37°C. (B) *C. albipunctata* eggs undergoing larval hatching at 37°C. (C) Representative images of a larva, pupa, and adult that successfully developed from eggs incubated at 37°C. (D) Egg hatching rate at 28°C and 37°C. (E) Larval survival rate at 28°C and 37°C. (F) Adult emergence rate at 28°C and 37°C. (Data in D–F are presented as mean ± SEM. **p < 0.01, ***p < 0.001).

The effect of mammalian physiological temperature on *C. albipunctata* development was assessed by comparing eggs incubated at 37°C with those maintained at the routine insect-rearing temperature of 28°C. Eggs were able to hatch at 37°C, and some individuals completed development to the pupal and adult stages (Fig 5A–C). However, quantitative analysis revealed that developmental performance was significantly reduced at 37°C compared with the 28°C controls. Specifically, the egg hatching rate decreased from 80.08% ± 2.90% at 28°C to 63.50% ± 2.58% at 37°C (Fig 5D, *p* < 0.01). Similarly, the larval survival rate dropped from 77.14% ± 1.66% to 52.53% ± 1.78% (Fig 5E, *p* < 0.001), and the adult emergence rate declined from 80.54% ± 1.71% to 59.35% ± 2.54% (Fig 5F, *p* < 0.001). These results indicate that while mammalian body temperature significantly impairs the developmental fitness of *C. albipunctata*, it does not completely block development.

### *In vitro* digestion of *C. albipunctata*

Upon exposure to *in vitro* simulated gastric and intestinal fluids (SGF/SIF), the developmental competence of *C. albipunctata* eggs was severely compromised (Table 2).

**Table 2:** Results of an *in vitro* digestion model for the treatment of larvae and eggs in *C. albipunctata.*

Subjects	*C. albipunctata* eggs	*C. albipunctata* larvae
Untreated control group	S&D^a^	-^c^
2-hour water treatment control group	S&D	S&D
4.5-hour water treatment control group	S&D	-^d^
SGF treatment group	All eggs were inactivated	Most of the larvae died
SIF treatment group	-^b^	A few of the larvae died
SGF + SIF treatment group	All eggs were inactivated	-^d^

^a^Surviving and developing into larvae or adults. ^b^Not applicable, as eggs must physiologically transit through the gastric phase prior to intestinal exposure *in vivo*. ^c^ Not applicable, as larval viability could be assessed via direct macroscopic observation, obviating the need for further developmental incubation. ^d^This experiment was not designed because most of the larvae died after the gastric digestion stage, and there was no need to simulate intestinal digestion to determine whether they were still alive after both the gastric and intestinal digestion stages; therefore, a 4.5-hour control experiment was not needed.

**Fig 6:**
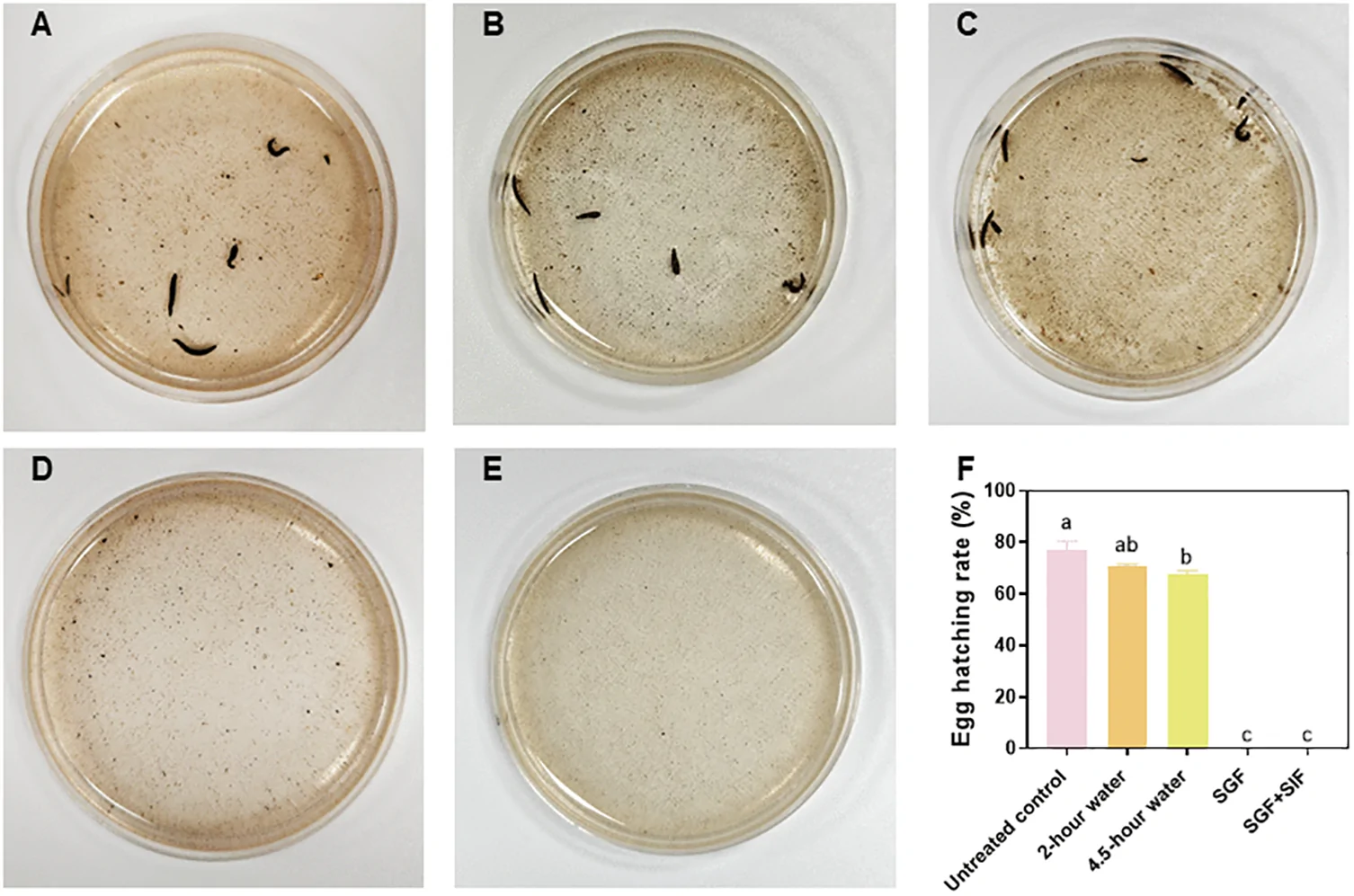
Hatching of *C. albipunctata* eggs after *in vitro* digestion. (A) *C. albipunctata* eggs in the untreated control group. (B) *C. albipunctata* eggs in the 2-h control group treated with water. (C) *C. albipunctata* eggs in the 4.5-h control group treated with water. (D) *C. albipunctata* eggs in the group treated with simulated gastric fluid (SGF). (E) *C. albipunctata* eggs in the group treated with simulated gastric solution + simulated intestinal solution (SGF + SIF). (F) Statistical analysis of the egg hatching rate across the five experimental groups. Different lowercase letters above the bars indicate statistically significant differences (*p* < 0.05, one-way ANOVA).

The INFOGEST-based *in vitro* digestion model was used to evaluate whether *C. albipunctata* eggs and larvae could tolerate simulated gastrointestinal fluids. In the egg hatching assay, macroscopic observations revealed that eggs in the untreated control (Fig 6A), 2-hour water control (Fig 6B), and 4.5-hour water control (Fig 6C) successfully hatched into active larvae. In striking contrast, exposure to simulated gastric fluid (SGF, Fig 6D) or sequential gastric and intestinal fluids (SGF + SIF, Fig 6E) completely inhibited hatching.

Statistical analysis of the hatching rates (Fig 6F) further confirmed these morphological observations. The egg hatching rate was robust in the untreated control group (76.80% ± 3.57%), and remained high in the 2-hour and 4.5-hour water-treated control groups (70.54% ± 1.11% and 67.76% ± 1.36%, respectively). However, the hatching rate dropped strictly to 0% after both SGF exposure and sequential SGF+SIF exposure. One-way ANOVA demonstrated that the simulated digestion treatments significantly abolished egg hatchability compared to all water-treated and untreated control groups (*p* < 0.05). These results firmly indicate that the gastric acid and enzymatic environment present an absolute barrier to the embryonic development and hatching of *C. albipunctata* eggs.

**Fig 7:**
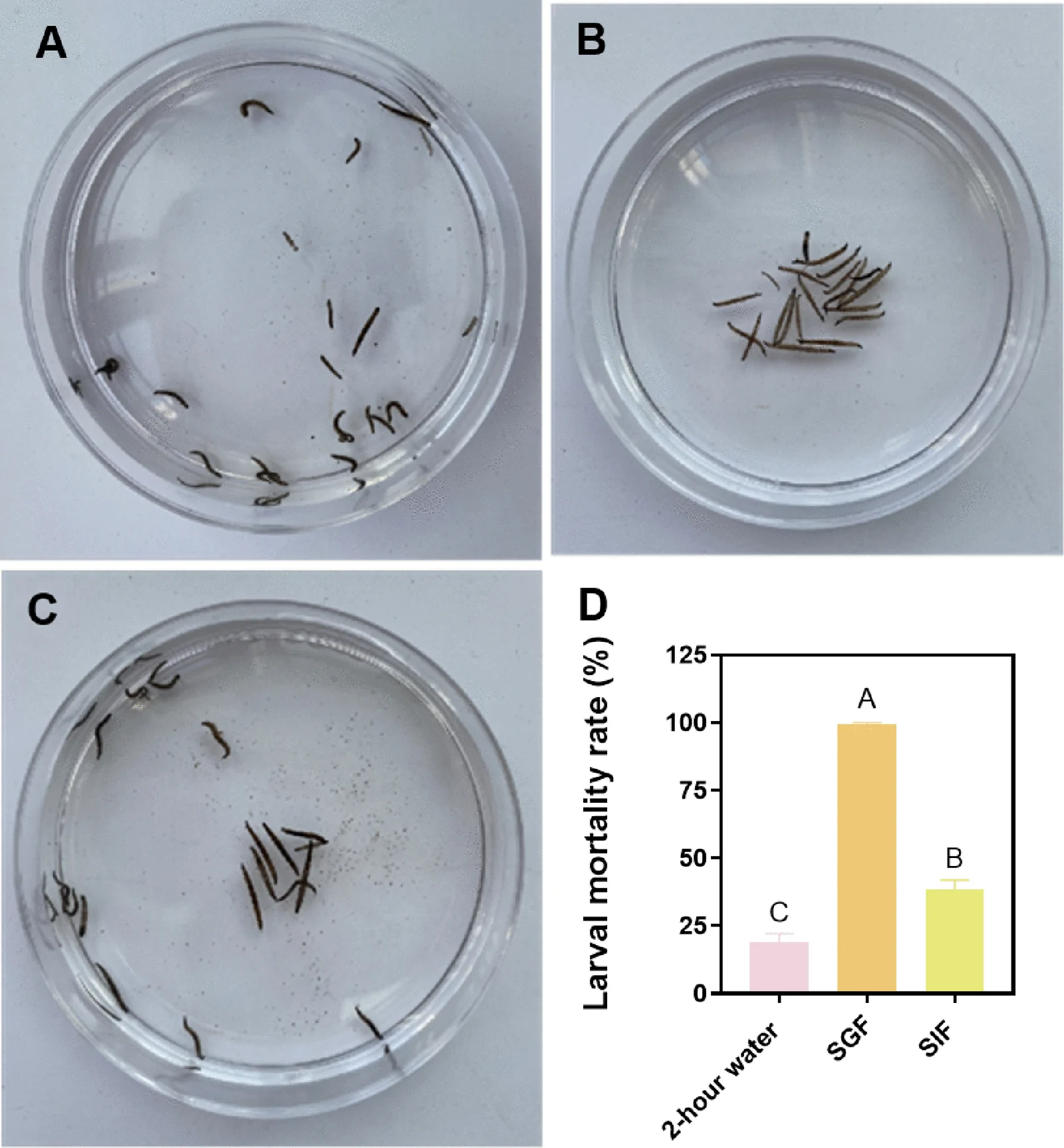
Effects of the *in vitro* digestion model on the survival of *C. albipunctata* larvae. (A) Larvae treated with water. (B) Larvae treated with simulated gastric solution. (C) Larvae treated with simulated intestinal solution. (D) Statistical analysis of the larval mortality rate across the different groups. Different uppercase letters above the bars indicate highly significant statistical differences (*p* < 0.01, one-way ANOVA).

Larval survival was also profoundly affected by simulated digestion. Macroscopic evaluation of larval morphology and activity revealed that larvae in the 2-hour water control group maintained intact locomotor competence and exhibited normal peripheral dispersion (Fig 7A). In stark contrast, SGF-exposed larvae displayed a rapid necrotic phenotype characterized by rigor mortis, centralized aggregation, complete loss of spontaneous movement, and an abolished response to tactile stimuli (Fig 7B). The SIF-treated cohort showed a bifurcated response: a portion of the larvae retained mobility, whereas others manifested locomotor arrest and tactile insensitivity (Fig 7C).

Quantitative statistical analysis of the larval mortality rates (Fig 7D) closely corroborated the morphological observations. The baseline natural mortality in the 2-hour water control group was relatively low (19.17% ± 3.01%). However, exposure to SGF induced a catastrophic physiological failure, driving the larval mortality rate to 99.17% ± 0.83%. Exposure to SIF alone also caused substantial damage, resulting in a mortality rate of 38.33% ± 3.58%. One-way ANOVA confirmed that the differences among all three groups were highly significant (*p* < 0.01). These data collectively demonstrate that standard gastric fluid acts as a strictly lethal barrier to *C. albipunctata* larvae, while intestinal fluid also inflicts significant physiological stress, rendering *in vivo* survival and colonization highly improbable.

**Fig 8:**
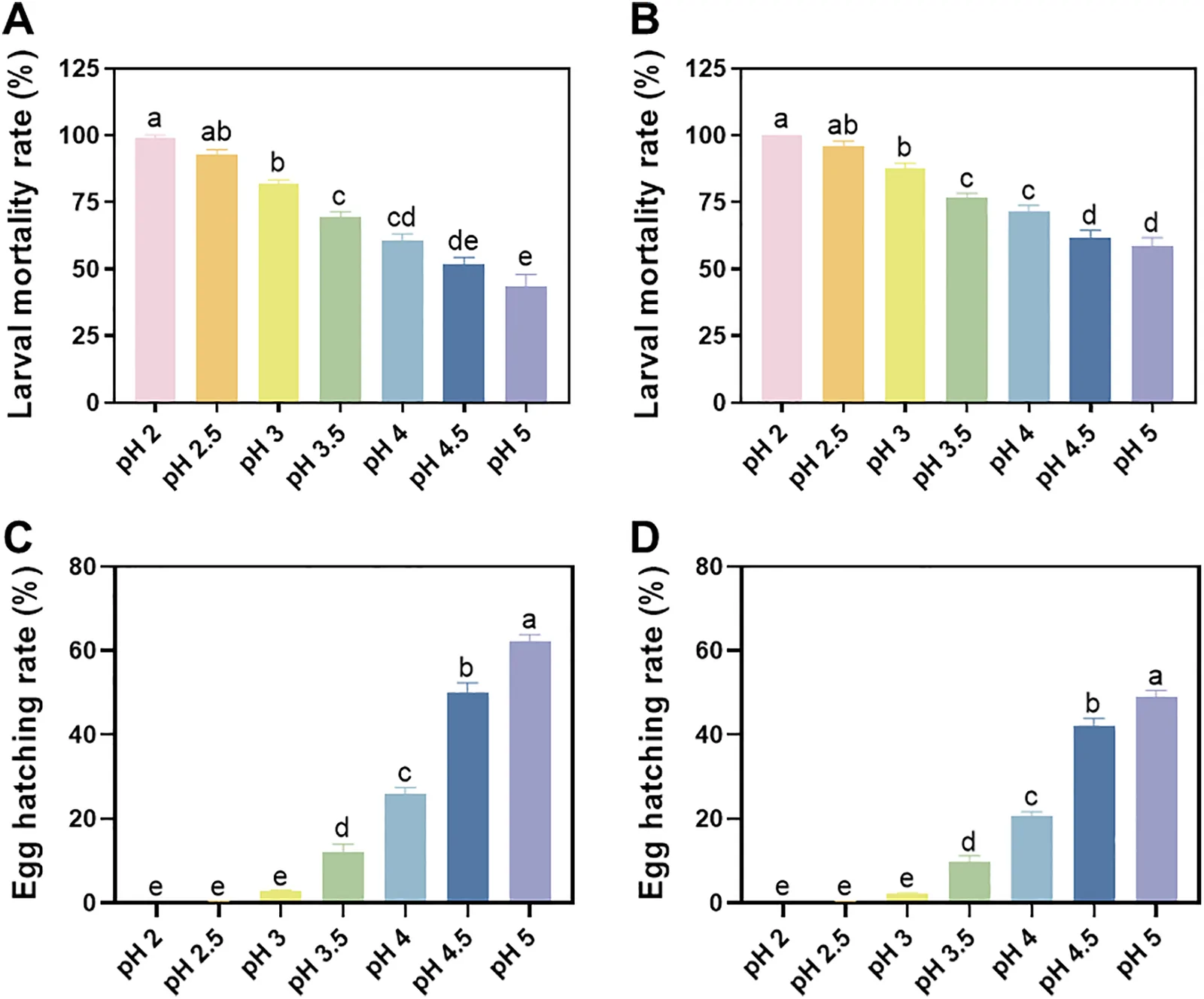
Effects of postprandial gastric pH gradients on the viability of *C. albipunctata* eggs and larvae. (A) Larval mortality rate after simulated gastric fluid (SGF) treatment at pH 2.0–5.0. (B) Larval mortality rate after sequential SGF and simulated intestinal fluid (SIF) treatment. (C) Egg hatching rate after SGF treatment at pH 2.0–5.0. (D) Egg hatching rate after sequential SGF and SIF treatment. Different lowercase letters indicate statistically significant differences among groups (*p* < 0.05, one-way ANOVA).

To evaluate whether the weakly acidic postprandial gastric environment could compromise the lethal effect of digestion, eggs and larvae were exposed to SGF across a pH gradient (pH 2.0–5.0). As expected, the elevation of pH significantly increased the survival of *C. albipunctata*. For larvae, the mortality rate decreased progressively from 99.17% ± 0.83% at pH 2.0 to 43.33% ± 4.77% at pH 5.0 after SGF treatment (Fig 8A), and sequential SGF+SIF treatment further induced mortality, reaching 58.33% ± 3.33% at pH 5.0 (Fig 8B). Similarly, egg hatching was inhibited at pH 2.0–2.5, but the hatching rate gradually increased as the pH rose, reaching 62.26% ± 1.55% at pH 5.0 in the SGF group (Fig 8C) and 48.96% ± 1.54% in the sequential SGF+SIF group (Fig 8D). These *in vitro* results indicate that a simulated postprandial increase in gastric pH partially attenuates the lethal effect of digestive fluids on *C. albipunctata*.

### Development of *C. albipunctata* in the digestive tract of mice

To validate the *in vitro* findings within a living animal model, BALB/c mice (*n* = 6) were gavaged with viable *C. albipunctata* eggs or first-instar larvae. In the egg-gavage group, no larvae, pupae, or adults were detected in fecal samples collected over seven consecutive days. In the larval-gavage group, no viable larvae or later developmental stages were detected in feces during the same observation period. Overall, fecal samples were examined, and all were negative for viable developmental stages of *C. albipunctata*.

**Fig 9:**
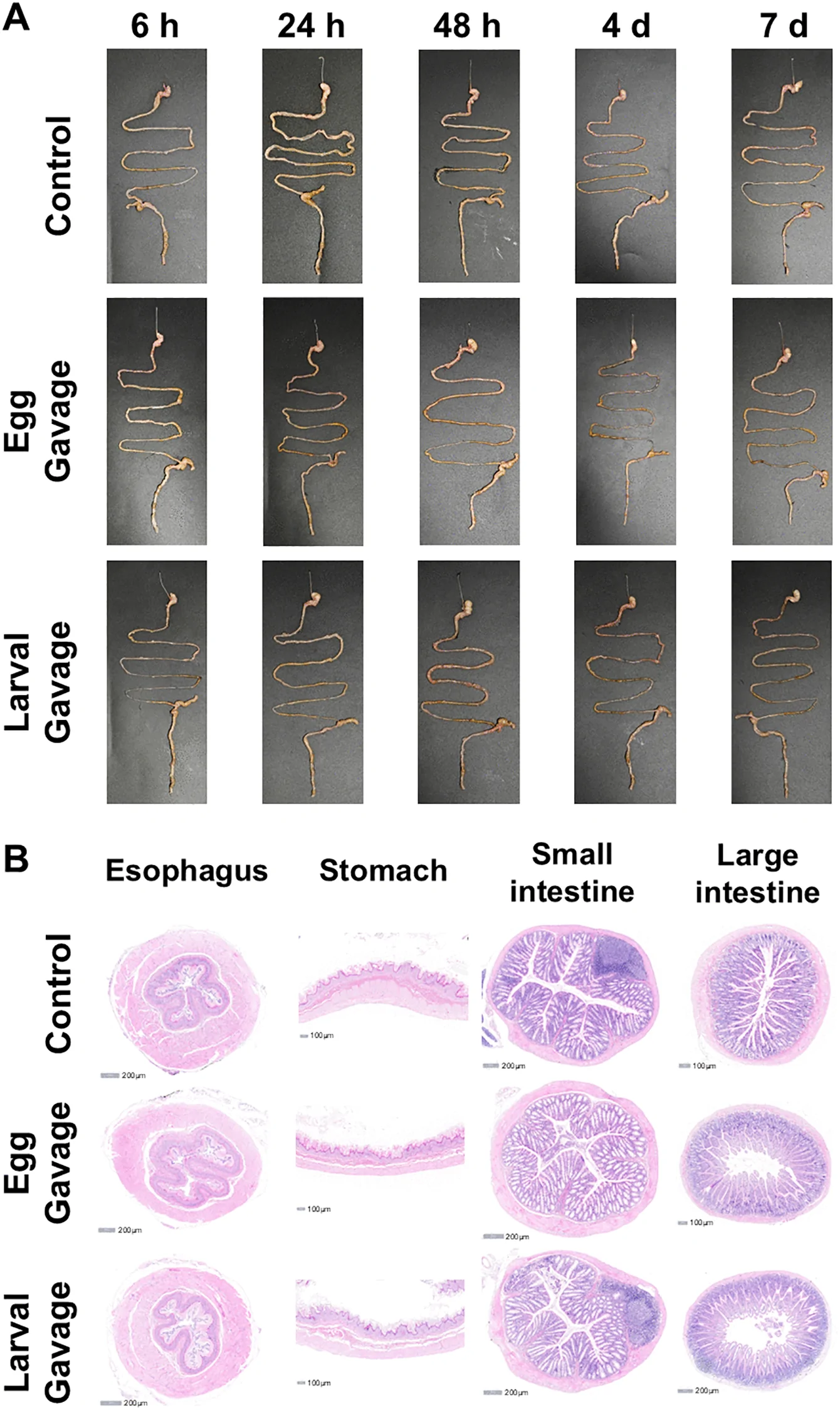
Macroscopic and histopathological evaluation of the murine gastrointestinal tract following oral gavage with *C. albipunctata.* (A) Time-course macroscopic observation of the extracted whole gastrointestinal tracts from the untreated control, egg-gavage, and larval-gavage groups at 6 h, 24 h, 48 h, 4 d, and 7 d post-inoculation. (B) Representative hematoxylin and eosin (H&E) stained sections of the esophagus, stomach, small intestine, and large intestine collected on day 7 post-gavage.

Time-point necropsy at 6 h, 24 h, 48 h, 4 d, and 7 d after gavage revealed no visible larvae, pupae, or adult stages in the esophagus, stomach, small intestine, or large intestine. No gastrointestinal obstruction, perforation, bleeding, or gross mucosal injury was observed in the egg-gavage, larval-gavage, or control groups (Fig 9A).

H&E staining of the esophagus, stomach, small intestine, and large intestine on day 7 showed preserved tissue architecture in all groups. No obvious epithelial disruption, ulceration, hemorrhage, or marked inflammatory cell infiltration was observed after egg or larval gavage compared with controls (Fig 9B). These results indicate that neither eggs nor first-instar larvae established detectable gastrointestinal colonization or caused obvious tissue injury in healthy BALB/c mice under the conditions tested.

## Discussion

According to the literature, human infestation by moth flies can involve multiple organ systems, including the gastrointestinal, nasal, and genitourinary tracts. Most of the reported cases occurred in regions with suboptimal socioeconomic and sanitary conditions [10–20,37,38]. Most diagnoses have relied primarily on patient self-reports, lacking conclusive evidence to rule out sample contamination. Furthermore, histopathologically confirmed cases of larval invasion into necrotic tissues remain exceedingly rare [38]. Furthermore, clinician awareness of delusional parasitosis is necessary when evaluating subjective symptoms [39–41]. While the rarity of authentic clinical cases suggests a negligible pathogenic potential for intestinal myiasis, rigorous experimental validation has been lacking. To address this clinical ambiguity, we systematically evaluated the pathogenicity of *C. albipunctata* through integrated *in vitro* simulations of the human digestive tract and *in vivo* murine models.

To ensure the reproducibility of our experimental system, we established a sustainable laboratory colony of *C. albipunctata*. Because morphological identification of immature stages can sometimes be challenging, we employed both morphological observation and mitochondrial COI barcoding to confirm the species identity, ensuring the accuracy of our downstream assays.

We initially investigated the effect of mammalian physiological temperature on *C. albipunctata* development. The findings indicated that while eggs could hatch and eventually mature into adults at 37°C, the developmental viability and overall activity were significantly reduced compared to their routine rearing temperature. Because mammalian body temperature alone did not completely abrogate development, temperature is not an absolute barrier. Thus, this phenomenon leaves open the possibility of moth fly parasitism in exposed non-gastrointestinal sites or surface wounds.

To determine whether *C. albipunctata* can survive inside the human digestive tract, we established an *in vitro* digestion model mimicking gastric and intestinal environments. The results revealed that standard simulated gastric fluid (SGF) and intestinal fluid (SIF) acted as formidable lethal barriers, entirely abolishing egg hatchability and inducing near-complete larval mortality. In considering postprandial physiology, we performed additional pH-gradient assays which confirmed that a weakly acidic environment (simulating postprandial conditions at pH 2.0–5.0) partially attenuates the lethality of gastric fluid, allowing a portion of eggs and larvae to survive *in vitro*. However, this *in vitro* survival does not easily translate to true intestinal myiasis *in vivo*. Physiologically, the postprandial elevation of gastric pH is transient; food intake stimulates continuous acid secretion, and prolonged gastric emptying ensures that ingested organisms eventually face a highly acidic environment. Furthermore, any survivors must subsequently endure the enzymatic actions of intestinal fluids (which caused additional mortality in our assays) and the extreme hypoxic environment of the mammalian gut.

The *in vivo* murine experiments robustly supported this physiological reasoning. To comprehensively evaluate the infectious potential, we administered both viable eggs and first-instar larvae via oral gavage. The mice in our study were fed ad libitum (non-fasted), simulating natural postprandial conditions. Despite this, continuous fecal observation over seven days revealed no shedding of live larvae or other developmental stages. To eliminate the possibility of missing rapid degradation or transient survival, we performed time-point necropsies (6 h, 24 h, 48 h, 4 d, and 7 d post-gavage) and histopathological H&E staining, none of which showed gross mucosal injury, inflammatory cell infiltration, or retained insect structures in either the egg-gavage or larval-gavage groups. These results confirm that even direct ingestion of live larvae fails to establish true tissue colonization in a healthy mammalian gastrointestinal tract.

The discrepancy between our negative experimental results and published human case reports may have several explanations. In settings with poor sanitation, eggs or larvae may colonize fecal samples after defecation, leading to misdiagnosis. Even if a small number of ingested eggs or larvae withstand digestion and are excreted alive, this phenomenon represents mechanical passage (pseudomyiasis) rather than true pathological tissue invasion. While our study primarily addressed gastrointestinal myiasis, the etiology of reported genitourinary and respiratory myiasis also warrants cautious evaluation. Given the anatomical barriers and the rarity of retrograde ascending infections in the human urogenital tract [2], it is highly improbable that *C. albipunctata* easily completes its life cycle in these environments.

This study has several limitations that should be acknowledged. First, the in vivo experiments utilized a relatively small sample size (*n* = 6 mice per group at each time point). Furthermore, given the inherent species differences in gastrointestinal physiology between mice and humans, murine models cannot fully substitute for human studies. The absence of non-human primate models or direct clinical validation remains a limitation. Second, healthy BALB/c mice do not fully represent human hosts with specific vulnerabilities, such as severe hypochlorhydria, gastrointestinal motility disorders, altered microbiota, or immunosuppression. Third, while we performed routine H&E histological assessments to evaluate tissue damage, deep immunological profiling or 16S microbiome sequencing was not conducted; thus, subtle host immune reactions or microbiome alterations cannot be entirely excluded. Finally, the potential of *C. albipunctata* to colonize extra-gastrointestinal systems requires separate specialized models.

In conclusion, our integrated *in vitro* and *in vivo* findings demonstrate that *C. albipunctata* eggs and larvae are highly vulnerable to digestive fluids and fail to establish intestinal colonization or induce tissue injury in healthy murine models. Present results suggest that many reported clinical cases may represent sample contamination or pseudomyiasis rather than true infection. This evidence provides a scientific foundation for differential diagnosis, helping clinicians prevent misdiagnosis and avoid unnecessary antiparasitic interventions.

## Supporting information

S1 Table Primers for amplification of the genomic DNA of moth fly larvae. (DOCX)

S2 Table The PCR conditions. (DOCX)

S1 File Raw image of the agarose gel electrophoresis shown in Fig 4. (PDF)

S2 File Raw experimental data. (XLSX)
