# Supplementary material for: Clogmia albipunctata fails to induce true intestinal myiasis: Evidence from in vitro and in vivo digestive models
Source: PLoS One. 2026 Aug 21;21(8):e0356742. doi: 10.1371/journal.pone.0356742 (PMC13496435; doi:10.1371/journal.pone.0356742)

Raw image of the agarose gel electrophoresis shown in Fig 4

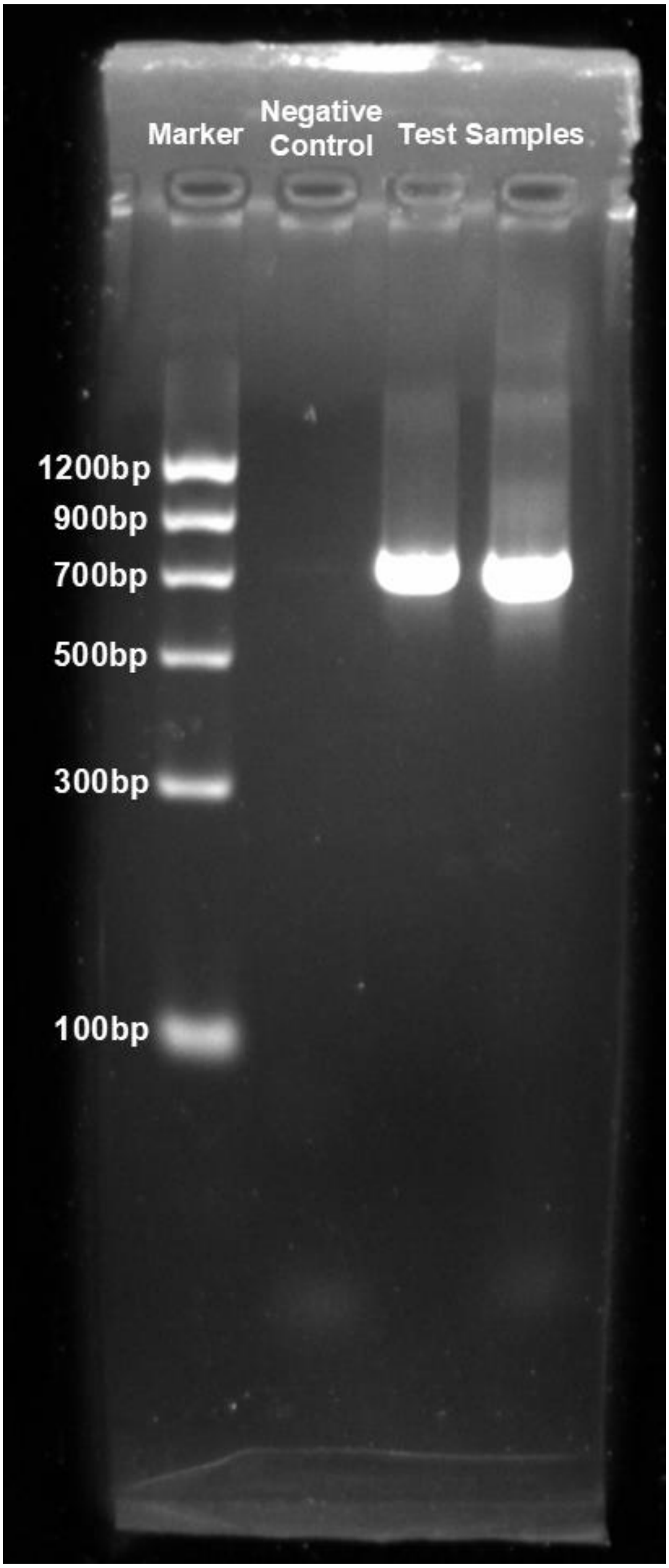

Supplement: Supplementary file 1 [file pone.0356742.s003.pdf]
