# Supplementary material for: Clogmia albipunctata fails to induce true intestinal myiasis: Evidence from in vitro and in vivo digestive models
Source: PLoS One. 2026 Aug 21;21(8):e0356742. doi: 10.1371/journal.pone.0356742 (PMC13496435; doi:10.1371/journal.pone.0356742)
Supplement: Supplementary file 2 [file pone.0356742.s001.docx]

**S1 Table. Primers for amplification of the genomic DNA of moth fly larvae**

| **Primer** | **Sequence (5’-3’)** |
| --- | --- |
| LCO1490 (Forward) | GGTCAACAAATCATAAAGATATTGG |
| HCO2198 (Reverse) | TAAACTTCAGGGTGACCAAAAAATCA |
