# Supplementary material for: Clogmia albipunctata fails to induce true intestinal myiasis: Evidence from in vitro and in vivo digestive models
Source: PLoS One. 2026 Aug 21;21(8):e0356742. doi: 10.1371/journal.pone.0356742 (PMC13496435; doi:10.1371/journal.pone.0356742)
Supplement: Supplementary file 3 [file pone.0356742.s002.docx]

**S2 Table. The PCR conditions**

| **PCR step** | **Temperature** | **Time** | **Repeat cycle** |
| --- | --- | --- | --- |
| Initial denaturation | 94℃ | 5 min | 1 |
| Denaturation | 94℃ | 30 s | 35 |
| Annealing | 45℃ | 50 s | 35 |
| Extension | 72℃ | 1 min | 35 |
| Final extension | 72℃ | 10 min | 1 |
| Hold | 4℃ | Forever | - |
